# An Updated Review on the Emerging Role of Indocyanine Green (ICG) as a Sentinel Lymph Node Tracer in Breast Cancer

**DOI:** 10.3390/cancers15245755

**Published:** 2023-12-08

**Authors:** Ioanna Akrida, Nikolaos V. Michalopoulos, Maria Lagadinou, Maria Papadoliopoulou, Ioannis Maroulis, Francesk Mulita

**Affiliations:** 1 Department of Surgery, General University Hospital of Patras, 26504 Rio, Greece; ioannaakrida@upatras.gr (I.A.); marlo@upatras.gr (I.M.); 2 4th Department of Surgery, Attikon University Hospital, Medical School, National and Kapodistrian University of Athens, 1 Rimini Street, Chaidari, 12462 Athens, Greece; nmichal@med.uoa.gr (N.V.M.); mpapadoliop@med.uoa.gr (M.P.); 3 Department of Internal Medicine, General University Hospital of Patras, 26504 Rio, Greece; mlagad@upatras.gr

**Keywords:** indocyanine green, ICG, fluorescence, sentinel lymph node biopsy, breast cancer

## Abstract

**Simple Summary:**

Dual lymphatic mapping using radioisotope and blue dye is the gold standard technique for performing sentinel lymph node biopsy (SLNB) in breast cancer. However, it is associated with significant risk for anaphylactic reactions to blue dye, and with difficulties related to the supply and usage of radioactive agents. Therefore, there is an imperative need for the development of novel sentinel lymph node tracers that are safe, cheap and easily available. Indocyanine green (ICG) fluorescence is the most promising among the innovative techniques for lymphatic mapping in breast cancer and it has been introduced in everyday clinical practice in several countries. The literature on the use of ICG for SLNB in breast cancer is constantly growing. This is an updated review of some recent studies that show how ICG could complement or even replace the conventional sentinel lymphatic mapping tracers.

**Abstract:**

Sentinel lymph node biopsy (SLNB) has become the standard of care for clinically node-negative breast cancer and has recently been shown by clinical trials to be also feasible for clinically node-positive patients treated with primary systemic therapy. The dual technique using both radioisotope (RI) and blue dye (BD) as tracers for the identification of sentinel lymph nodes is considered the gold standard. However, allergic reactions to blue dye as well as logistics issues related to the use of radioactive agents, have led to research on new sentinel lymph node (SLN) tracers and to the development and introduction of novel techniques in the clinical practice. Indocyanine green (ICG) is a water-soluble dye with fluorescent properties in the near-infrared (NIR) spectrum. ICG has been shown to be safe and effective as a tracer during SLNB for breast cancer and accumulating evidence suggests that ICG is superior to BD and at least comparable to RI alone and to RI combined with BD. Thus, ICG was recently proposed as a reliable SLN tracer in some breast cancer clinical practice guidelines. Nevertheless, there is lack of consensus regarding the optimal role of ICG for SLN mapping. Specifically, it is yet to be determined whether ICG should be used in addition to BD and/or RI, or if ICG could potentially replace these long-established traditional SLN tracers. This article is an updated overview of somerecent studies that compared ICG with BD and/or RI regarding their accuracy and effectiveness during SLNB for breast cancer.

## 1. Introduction

The surgical management of the axilla in breast cancer patients has undergone major changes over the last three decades. In clinically node-negative breast cancer, sentinel lymph node biopsy (SLNB) has replaced axillary lymph node dissection (ALND) and has become the standard of care for axillary staging. The influential ACOSOG Z0011 clinical trial has led to further de-escalation of the surgical treatment of the axilla in breast-conserving patients with T1-2 breast cancer and 1–2 positive sentinel lymph nodes (SLNs) [1]. In this subgroup of sentinel node-positive patients, ALND can be safely avoided, thus sparing the patients from the significant long-term side effects caused by ALND. AMAROS, another practice-changing trial, included both breast-conserving and mastectomy patients and indicated that axillary radiotherapy is a safe alternative to ALND in T1-2 patients with 1-2 positive SLNs [2].

In favor of de-escalation of axillary surgery in breast cancer, the American Society of Breast Surgeons suggests that SLNB can be omitted in women aged over 70 years old with clinically T1-2N0, hormone receptor positive and HER2 negative tumors [3]. Notably, SLNB was recently introduced in clinical practice also for clinically node-positive patients who become clinically node-negative after neoadjuvant chemotherapy (NACT) [4]. Thus, indications for SLNB have expanded beyond patients presenting with clinically node-negative early breast cancer.

Since the first reports on the feasibility of the radio-guided and dye-directed SLNB for breast cancer, by Krag in 1993 [5] and by Giuliano in 1994 [6], respectively, the technique of SLNB has undergone significant evolution. Currently, the dual mapping technique combining both a radioisotope and a blue dye is considered the gold standard technique that provides the highest sentinel lymph node identification rate and the lowest false negative rate (FNR) [7].

Blue dyes used for SLNB include isosulfan blue (popular in USA), an isomer of patent blue, as well as methylene blue, which is more widely available and less expensive. One significant disadvantage of the use of isosulfan blue and patent blue as tracers during SLNB is the rate of allergic and anaphylactic reactions, estimated to range from 0.06% to 2.7% [8,9]. Life-threatening allergic reactions to methylene blue are rare; however, semi-permanent skin tattooing is not uncommon and cases of skin necrosis after intradermal injection have also been reported [8,9]. Like blue dyes, radioisotopes do not come without drawbacks. The use of Technetium 99m (^99m^Tc), the preferred radiotracer in SLNB for breast cancer (most commonly ^99m^Tc-sulfur colloid in USA and ^99m^Tc-nanocolloid human serum albumin in Europe), is expensive, requires anuclear medicine facility, correlates with radiation exposure to both the patient and the operating theater personnel and is also associated with logistics issues regarding both the supply and the discharge of radioactive agents.

The disadvantages of the traditional SLNB tracers have led to the need for research on novel agents for intraoperative lymphatic mapping in breast cancer. Indocyanine green (ICG) is a relatively nontoxic dye used in medicine since 1950. ICG was originally used to test liver function and cardiac output by measuring ICG blood levels and it was subsequently used in ophthalmic angiography [10]. The discovery of the fluorescent properties of the molecule of ICG, which is capable of absorbing light and emitting in the near-infrared (NIR) spectrum, as well as the recent progress of imaging NIR technologies, expanded the clinical usage of ICG in many aspects of surgery, including SLN mapping, tumor identification, lymphedema management and the evaluation of microvascular circulation during reconstructive surgery [11,12].

There is growing evidence to support that SLNB in breast cancer using ICG is safe and feasible. The literature comparing ICG with BD and/or IR is constantly expanding and, recently, some guidelines from Europe and Asia included ICG as a tracer appropriate for performing SLNB in breast cancer. This article is an updated review of some recent studies comparing ICG with BD and/or RI as SLNB tracers in breast cancer. This review is based on PubMed searches (accession date 21 September 2023) performed for the present manuscript, using the terms [(“ICG” OR “indocyanine green”) AND “sentinel lymph node” AND “breast cancer”] which yielded a total of 135 results. The limit of publication dates was within the last 5 years. The titles and abstracts of the above articles were screened and 38 relevant clinical studies were selected and included in this review. Furthermore, our PubMed search using the terms [(“ICG” OR “indocyanine green”) AND “sentinel lymph node” AND “breast cancer” AND “(meta-analysis OR metaanalysis”)], without any limit of publication date, yielded results whose titles and abstracts were screened and 10 relevant meta-analyses articles were selected and included in this review.

## 2. Studies Using ICG and BD as SLNB Tracers

One of the main drawbacks of using radioisotopes as SLNB tracers is the issue of reduced availability of Technetium 99m which regularly suffers from supply chain shortage [13]. Particularly in low- and middle-income countries with inadequate access to radioactive molecules, the surgical treatment of patients eligible for SLNB commonly relies on blue dye alone, which is inferior to both Technetium 99m alone as well as to the standard dual RI + BD technique [14]. Therefore, several authors investigated the efficacy of SLNB mapping using BD, the traditional SLNB tracer, in combination (+) with ICG, a novel and promising SLNB tracer (Table 1).

Three recent prospective randomized clinical trials evaluated the use of ICG (alone or in combination with BD), in comparison with the use of BD alone. Coibion et al. randomized 240 cN0 early breast cancer patients to SLNB using either ICG or BD as a tracer and resulted in an identification rate (IR) of 100% and 97.5% for ICG and BD, respectively, with a mean number of excised SLNs of 3.6 for ICG and 3 for BD. Therefore, the authors concluded that ICG was not inferior to BD [15]. Zhang et al. randomized 415 cT1-2N0 patients to either ICG combined with BD, or to BD alone, and found that the detection rate was 89.7% with BD alone compared with 96.9% with ICG combined with BD, and this difference was significant. The authors also reported a significant difference regarding the false negative rate (FNR) that was in favor of using ICG and BD, instead of BD alone [16]. In another Chinese study. Qin et al. randomized 180 patients to receive ICG combined with BD, BD alone or BD combined with carbon nanoparticles, and have shown that ICG combined with BD is comparable to BD alone regarding the identification rates but is characterized by a significantly larger number of SLNs excised [17].

Several prospective single-arm observational studies included patients submitted to SLNB directed by both ICG and BD, and investigated the accuracy of ICG compared with the accuracy of BD. The authors of these studies reported an IR of 70% to 93% for BD, in contrast to a detection rate of 89.2% to 100% for ICG, and this difference was statistically significant [18,19,20,21,22,23]. It is noteworthy that the mean number of SLNs was reported to be significantly larger when ICG was used as a tracer compared with BD. One prospective comparative non-randomized trial from China enrolled 523 cN0 early breast cancer patients and compared the use of BD alone with the use of ICG combined with BD. In accordance with the aforementioned studies, the authors concluded that the IR of ICG is 99.2%, significantly higher than that of BD (93.3%), whereas the mean number of SLNs retrieved is significantly higher when using ICG [24].

Several retrospective single-arm studies reviewed the clinical data of patients that have undergone SLNB using ICG combined with BD as SLN mapping tracers. These studies reported a detection rate of 97.4% to 100% for the dual ICG + BD method, with an FNR of approximately 7% for this combined method [25,26,27]. Interestingly, Xu et al., in a retrospective analysis on 127 breast-conserving patients who met the ZOO11 eligibility criteria, found that 53/127 patients had SLNs positive for ICG and negative for BD and, more importantly, 16/127 patients had metastatic lymph nodes positive for ICG and negative for BD [26]. Two large retrospective single-arm studies, both of Chinese origin, enrolled 1574 and 1061 patients, respectively, treated with dual ICG- and BD-directed SLNB and reported an identification rate of over 99.5% and a postoperative regional axillary recurrence in approximately 0.7% of the patients with negative SLNs, after approximately 5 years of follow up [25,28].

In accordance with the above studies, two retrospective comparative studies confirmed the superiority of the combined ICG and BD method, over the use of BD alone. Yang et al. [29] compared the use of ICG combined with BD, with the use of BD alone, whereas Takemoto et al. [30] compared the ICG combined with BD method, with the use of ICG alone. They both reported a significantly higher IR for the dual ICG and BD mapping (98.5% and 96.4%, respectively), compared with the detection rate of BD alone (91.5% and 83.7%, respectively).

**Table 1:** Studies using ICG and BD as SLNB tracers.

Study	Country	Tracer	Type of Study	No. of Patients	Eligibility Criteria	Results	Reference
Coibion et al., 2022	Belgium	ICG vs. BD	prospective randomized trial	240	cN0 early breast cancer	IR of ICG 100% IR of BD 97.5% Mean number of SLNs for ICG 3.6 and for BD 3	[15]
Lin et al., 2020	Taiwan	ICG + BD	prospective observational study	39	cN0 early breast cancer	IR of ICG 97.5% IR of BD 70% Mean number of SLNs for ICG 2.1 and for BD 0.93 (*p* < 0.001) Obesity significantly reduces IR for ICG (in BMI < 30 IR 100% and in BMI > 30 IR 75%)	[22]
Ng et al., 2022	UK	ICG + BD	prospective observational study	239	all SLNB eligible patients (of whom 24 post-NAC and 35 post-NET)	IR of ICG 98.6% IR of BD 85.1% Nodal detection rate post-NAC 87.5% (21/24) and post-NET 100% (35/35) ICG is effective regardless of BMI	[21]
Pinto el al. 2022	Portugal	ICG + BD	prospective observational study	37	cN1 after NAC (towards SLNB-TAD with ultrasound guided excision of the clipped node)	IR of ICG 89.2% IR of BD 85.5%	[20]
Qin et al., 2019	China	ICG + BD, BD, BD + CNP	prospective randomized trial	180	cTis-3N0	IR of ICG + BD 100% IR of BD 96.7% IR of BD + CN 98.3% (*p* = 0.362) Mean number of SLNs for ICG + BD 3.4, for BD 1.7 and for BD + CN 2.4 (*p* < 0.001)	[17]
Shen et al., 2018	China	ICG + BD vs. BD	prospective non-randomized trial	523	cT1-2N0	IR of ICG 99.2% IR of BD 93.3% (*p* < 0.001) Mean number of SLNs for ICG + BD 3.7 and for BD 3.2 (*p* = 0.004)	[24]
Takemoto et al., 2018	Japan	ICG + BD vs. ICG	retrospective study	202	cT1-2N0	IR of ICG + BD 96.4% IR of BD 83.7% IR of ICG signficantly lower for BMI > 25	[30]
Wang et al., 2021	China	ICG + BD	prospective single-arm study	60	cN0 early breast cancer	IR of ICG + BD 100% IR of BD 87% Mean number of SLNs for ICG + BD 3, for BD 2 (*p* < 0.05)	[18]
Wang et al., 2021	China	ICG + BD	retrospective study	1061	cTis-3N0	IR of ICG + BD 99.6% 94.1% of positive nodes could be identified with ICG + BD Ipsilateral axillary recurrence 0.64% in patients with negative SLNs after 5.6 years of follow up	[28]
Wang et al., 2020	China	ICG + BD	prospective non-randomized trial	70	cT1-3N0	IR of ICG 100% IR of BD 93% Mean number of SLNs for ICG 3.5, for BD 2.4 (*p* < 0.05)	[19]
Xu et al., 2022	China	ICG + BD	retrospective study	156	cN0 stage I, II breast cancer	IR of ICG + BD 97.4% IR of BD 84.6% IR of ICG 92.9% FNR of ICG + BD 7.3%, BD 10.9% and ICG 9.1%	[27]
Xu et al., 2022	China	ICG + BD	retrospective study	127	breast-conserving patients who underwent SLNB and met the criteria of Z0011 trial	53/127 patients had SLNs with ICG(+)/MB(−) phenotype 16/127 patients had metastatic SLNs with ICG(+)/MB(−) phenotype	[26]
Yang et al., 2023	China	ICG + BD vs. BD	retrospective study	300	cT1-2N0	IR of ICG + BD 98.5% IR of BD 91.5% (*p* = 0.007) Mean number of SLNs for ICG + BD 3.1, for ICG 3 and for BD 2.6 (*p* < 0.05)	[29]
Yang et al., 2023	China	ICG + BD	retrospective study	1574	cTis-2N0	IR of ICG + BD 99.7% Postoperative regional lymph node recurrence rate is 0.7% in patients with negative SLNs (4.7 years of follow up)	[25]
Yuan et al., 2019	China	ICG + BD	observational study	312	cT1-3N0	IR of ICG + BD 100% IR of ICG 93.1% IR of BD 89.5% FNR of ICG + BD, ICG and BD are 11.2%, 11.1% and 16.7%, respectively	[23]
Zhang et al., 2021	China	ICG + BD vs. BD	randomized controlled trial	415	cT1-2N0	IR of ICG + BD 96.9% IR of BD 89.7% FNR of ICG + BD and BD is 7.3% and 10.5%, respectively (*p* > 0.05)	[16]

ICG: indocyanine green, BD: blue dye, IR: identification rate, SLNs: sentinel lymph nodes, SLNB: sentinel lymph node biopsy, NAC: Neoadjuvant chemotherapy, NET: neoadjuvant endocrine therapy, TAD: targeted axillary dissection, CN: carbon nanoparticle, FNR: false negative rate.

Therefore, the existing data suggest that SLN mapping in breast cancer with the use of ICG is superior to the use of BD alone but is associated with an increased number of SLNs excised. Furthermore, the combined ICG +BD method yields excellent results, raising the question whether ICG could potentially replace the radioisotope in the gold standard dual RI + BD technique.

## 3. Studies Using ICG and RI as SLNB Tracers

The success of the Technetium 99m-guided SLNB technique is superior to that of BD alone, and except for the highly experienced centers, the success of the gold standard isotope plus blue dye dual method is superior to the success of either method alone [3]. In order to determinate whether ICG could potentially replace the radiotracer in the SLN mapping for breast cancer axillary staging, studies comparing the accuracy of ICG with that of RI were required. Furthermore, the use of BD, especially that of isosulfan blue, is characterized by an uncommon but not rare risk of anaphylactic reactions. Thus, several studies included patients that have undergone SLNB using ICG in combination with RI for SLN mapping, without the concurrent use of BD (Table 2).

Two prospective randomized clinical trials examined the use of ICG plus RI and compared it with the use of RI alone. A French study by Vermersch et al. enrolled 99 cN0 early breast cancer patients and found that the retrieved SLNs were ICG positive in 92.6% of the patients, and RI positive in 85.2% of the patients [31]. Furthermore, the authors reported a significantly higher number of SLNs retrieved in the ICG plus RI group of patients, in comparison with the patients that received Technetium 99m only directed at SLNB (2.14 and 1.77 nodes, respectively). The second randomized trial, a Korean study by Jung et al., included patients in the post NACT setting and reported an identification rate of 98.3% for the combined ICG plus RI method, compared with 93.8% for the RI alone technique, but this difference was not statistically significant [32]. In this study, the authors did not find a significant difference regarding the number of SLNs excised.

Various prospective and retrospective single-arm studies used ICG combined with RI as SLN tracers and compared the accuracy of ICG alone or combined with RI with that of RI alone. Some of these investigators reported an IR of over 95% for ICG and concluded that ICG is not inferior to the most commonly used radiotracer method [33,34,35,36,37,38]. It is noteworthy that two studies, one from the Netherlands and another from the UK, reported that ICG is superior to RI, with a detection rate of 73.4% to 86.7% for RI, and 96.1% to 98.1% for ICG [39,40]. On the contrary, one French study reported an IR of 82% for ICG, significantly lower than that of the RI (96.7%) [41]. Interestingly, most of these studies reported excellent results for the combined ICG plus RI method, with a detection rate ranging from 98% to 100% [32,34,35,36,37,40].

The concordance between the ICG and the traditional isotope technique was examined in most of these studies and was estimated to be approximately 80%. Specifically, a Korean study by Kang et al. found that 83% of the retrieved SLNs were identical regarding the uptake of ICG and RI, with a discordance rate of 17.7% [42]. However, in respect of the first SLN, the discordance rate was estimated to be 6.3%. Accordingly, the studies by Dumitru et al., Vermersch et al., Valente et al. and Mazouni et al. [31,38,40,41] reported that the concordance rate between ICG and RI was 71.4%, 78.7%, 81% and 82%, respectively. It should be noted that the discordance between ICG and RI in detecting metastatic lymph nodes was reported to be 11.3% [34], which means that approximately one out of ten patients would be false negative when using ICG or RI alone. This implies that the discrepancies in the two methods, ICG and RI, compensate for each other’s false negatives. Thus, one could hypothesize that ICG may not be an optimal tracer to be used alone for SLN mapping in breast cancer.

**Table 2:** Studies using ICG and RI as SLNB tracers.

Study	Country	Tracer	Type of Study	No. of Patients	Eligibility Criteria	Results	Reference
Bargon et al., 2022	Netherlands	ICG + RI	prospective single-arm noninferiority study	102	cT1-2N0 breast-conserving patients	IR of ICG 96.1% IR of RI 86.7% IR of pathological SLNs 86.7% for both ICG and RI	[39]
Dumitru et al., 2022	UK	ICG + RI	prospective observational study	79	cTis-2N0	IR of ICG + RI 98.7% IR of ICG 98.1% IR of RI 73.4% Concordance rate 71.4% between ICG and RI [90% of SLNs were ICG(+) and 73.4% of SLNs were RI(+)]	[40]
Fregatti et al., 2021	Italy	ICG + RI	prospective study	54	cT1N0	IR of ICG 96.3% IR of RI 100% ICG is cost-effective if the cost of the device is not included (with the addition of the cost of the device, when 118 patients undergo SLNB by ICG, there is a net saving of 254 euro per patient)	[33]
Jimbo et al., 2022	Japan	ICG + RI	retrospective study	338	cT0-3,N0	IR of ICG + RI, ICG, RI was 99.7%, 96.4% and 91.7%, respectively (not significant). However, 411.3% discordance between ICG and RI in detecting metastatic disease (i.e., 38/338 patients would be false negative with ICG or RI alone)	[34]
Jung et al., 2019	Korea	ICG + RI vs. RI	phase 2, prospective, single-center, randomized trial	130	cT0-4N0-3M0 patients in post NACT setting	IR of ICG + RI 98.3% IR of RI 93.8% Average number of SLNs 2.2 with ICG + RI and 1.9 with RI alone (not significant)	[32]
Kang et al., 2022	Korea	ICG + RI	prospective comparative study	78	cTis-2,N0-1	83–84% of retrieved SLNs were identical in the uptake of ICG and RI. Discordance between ICG and RI 17.7% (but regarding the first SLN, the discordance rate is 6.3%)	[42]
Mazouni et al., 2018	France	ICG + RI	prospective study	122	cT0-2N0	IR of ICG + RI 98.3% IR of ICG 82% IR of RI 96.7% Concordance between ICG and RI 82% Obesity and SLN macrometastasis strongly correlate with ICG failure	[41]
Ngo et al., 2020	France	ICG + RI	prospective clinical trial	77	cT1-2N0	IR of ICG + RI 99% IR of ICG 96% IR of RI 93% No difference in FNR Mean number of SLNs for ICG + RI 2.7, for ICG 2.3 and for RI 2.3 (not significant)	[35]
Papathemelis et al., 2018	Germany	ICG + RI	retrospective single-arm single-center study	104	all SLNB eligible patients (including 19 after NACT)	IR of ICG + RI 100% IR of ICG 98% IR of RI 98% FNR for ICG was 0% and for RI was 4.8% all 10 post NACT patients were ICG(+)	[36]
Staubach et al., 2022	Germany	ICG + RI	retrospective double-arm study	161	cN0 early breast cancer vs. patients receiving SLNB in the post NACT setting	For primary SLNB: IR of ICG, RI and ICG + RI were 94.3%, 96.2% and 98.1%, respectively. For SLNB post NACT: IR of ICG 95.4% and IR of RI 95.4%	[37]
Valente et al., 2018	USA	ICG + RI	prospective clinical trial	92	cT1-2N0	81% of SLNs were ICG(+)/RI(+) 14% of SLNs were ICG(+)/RI(−) 5% of SLNs were ICG(−)/RI(+) ICG is equivalent to RI regarding IR	[38]
Vermersch et al., 2019	France	ICG + RI vs. RI	randomized controlled trial	99	cN0 breast cancer	SLNs were ICG(+) in 92.6% of patients, RI(+) in 85.2% and both ICG+RI(+) in 78.7% Mean number of SLNs for ICG+RI 2.14 and for RI 1.77 (*p* = 0.09)	[31]

ICG: indocyanine green, IR: identification rate, SLNs: sentinel lymph nodes, SLNB: sentinel lymph node biopsy, NACT: neoadjuvant chemotherapy.

## 4. Studies Using ICG, RI and BD as SLNB Tracers

Based on the above data, it is evident that ICG is a better SLNB tracer for breast cancer, compared with the use of BD alone. Furthermore, ICG is probably comparable to the use of RI alone, whereas both the dual ICG + BD and the dual ICG+ RI techniques provide excellent results. Therefore, in search of the optimal role of introducing ICG widely into the clinical practice of SLNB, many authors conducted clinical trials on SLNB eligible patients, using all three tracers ICG, BD and RI in different combinations (Table 3).

Three studies compared the effectiveness of the combined ICG + BD method, with that of the gold standard dual RI + BD technique. Two randomized controlled trials enrolled SLNB eligible early breast cancer patients to receive either ICG + BD, or RI + BD directed axillary staging and did not report statistically significant difference regarding the identification rates and the FNR [43,44], but with a trend towards increased number of SLNs retrieved when the ICG + BD method is used. These results are in accordance with a retrospective analysis of clinically node-negative early breast cancer patients submitted to SLNB, which reported an identification rate of 97% and 95% for the ICG + BD and the RI + BD methods, respectively, a difference that was not significant [45]. We also identified one study, the so called GREENORBLUE trial, that compared the accuracy of the gold standard RI + BD method with that of RI + ICG-directed SLNB. The authors concluded that the ICG + RI technique is a safe and effective alternative to the gold standard dual method. It should be noted that intraoperative anaphylaxis occurred in 2 out of 150 patients in the Technetium 99m plus patent blue V dye group of patients [46].

Several single-group studies examined the effectiveness of ICG as SLNB tracer, by evaluating the clinical data of patients that have undergone SLNB directed by all three tracers, ICG + BD + RI. These studies reported an IRs of 91.4% to 96% for the use of ICG [47,48,49,50]. Furthermore, Somashekhar et al. who analyzed the data of 100 clinically node-negative breast cancer patients who received ICG + BD + RI guided SLNB, concluded that the detection rate of ICG was 96%, not significantly different than the IR = 94% for the dual BD + RI method. Another investigator who enrolled 182 clinically node-negative patients, as well as patients in the post NACT setting, to receive SLNB with all three tracers, found that the ICG + BD method yielded IR = 100%, not significantly different than the IR = 98.9% for the standard BD + RI technique [51].

Finally, one large retrospective analysis on 1521 Indian patients who underwent SLNB with ICG + BD or RI + BD, or RI alone or BD alone found that the combined ICG + BD method correlates with the significantly higher IR of 98%, compared with the other techniques, as well as with significantly increased mean number of SLNs excised [52].

Therefore, the results of the aforementioned studies suggest that both the combined ICG + BD method and the ICG + RI technique are not inferior regarding their success, compared with the gold standard RI + BD SLNB mapping. Finally, it should be noted that, like conventional tracers, ICG has some disadvantages. One of them is the issue of cost-effectiveness. Suhani et al., via one randomized controlled trial, compared the use of ICG + BD, with the standard dual RI + BD method. They estimated that the cost of ICG plus BD is USD 175, whereas that of RI plus BD is USD 36, when the cost of the NIR imaging system is included [44]. However, other studies on the use of ICG, concluded that the cost of ICG drops below USD 50 when the imaging system equipment is not included [49,52]. Evidence from a study on the combined ICG plus RI method suggests that ICG is indeed cost-effective if the cost of the device is not included, but with the addition of the cost of the device, ICG becomes cost-effective after 118 SLNB procedures [33]. Another limitation of ICG may be the fact that ICG contains iodine. Therefore, it must be used with caution in patients with iodine or contrast allergy. In addition, ICG may be less effective in obese patients, since ICG can be visualized up to a depth of 1 cm.

**Table 3:** Studies using ICG, BD and RI as SLNB tracers.

Study	Country	Tracer	Type of Study	No. of Patients	Eligibility Criteria	Results	Reference
Agrawal et al., 2020	India	ICG + BD vs. RI + BD	retrospective analysis of prospectively collected data	207	cN0 early stage breast cancer	IR of ICG + BD 97% IR of RI + BD 95% (*p* = 0.72) Mean number of SLNs identified 2.73 for ICG + BD and 3.17 for RI + BD (*p* = 0.07) Average cost for ICG + BD USD 40 and for RI + BD USD 130	[52]
Agrawal et al., 2022	India	ICG + BD vs. BD vs. RI vs. RI + BD	retrospective analysis of prospectively collected data	1521	95% cN0 breast cancer and 5% in post NACT setting	IR of ICG + BD 98% IR of RI + BD 96% IR of BD 94% IR of RI 93.5% (*p* = 0.004) Mean number of SLNs for ICG + BD 3.4 and for RI + BD 2.8 (*p* < 0.001)	[45]
Chirappapha et al., 2020	Thailand	ICG + BD + RI	prospective study	23	after NACT	IR of ICG, BD and RI was 95.23%, 85.71% and 85.71% IR of ICG + BD 100% IR of RI + BD 95.23% FNR for ICG, BD, RI were 10%, 30% and 40%, respectively, and for ICG + BD + RI < 10%	[50]
Hua et al., 2022	China	ICG + BD + RI ICG + BD	retrospective study	194	cN0	IR of ICG 95.5% IR of RI 95.5% IR of BD 86.4% IR of ICG + RI + BD 100% FNR no significant differences	[47]
Jin et al., 2022	China	ICG + BD + RI	prospective self-controlled	182	cN0 breast cancer (152/182) and after NACT (30/182)	IR of ICG + BD 100% IR of RI + BD 98.9% Significantly more SLNs detected with ICG + BD vs. RI + BD Similar IRs between the cN0 and the post NACT group	[51]
Nguyen et al., 2022	Australia	ICG + RI vs. BD + RI	prospective cohort of patients undergoing ICG + RI SLNB, compared with retrospective cohort of standard BD + RI SLNB	300	cN0 early breast cancer	ICG + RI is an effective and safe alternative to standard BD + RI In the BD + RI group there were 2 cases of anaphylaxis and 2 cases of skin tattooing	[47]
Somashekhar et al., 2020	India	ICG + RI + BD	non-randomized prospective observational stusy	100	cN0 early breast cancer	IR of ICG 96% IR of RI + BD 94% False negative rates for ICG 3.1% and for RI + BD 6.2% Cost of RI + BD USD 350 per patient, whereas <USD 50 for ICG (imaging system instrument not included)	[49]
Suhani et al., 2023	India	ICG + BD vs. RI + BD	randomized controlled trial	70	cTis-2N0	IR of ICG + BD 100% IR of RI + BD 91.4% (*p* = 0.07) Mean number of SLNs for ICG + BD 4 and for RI + BD 3 (*p* = 0.048) Cost of ICG + BD USD 175 and for RI + BD USD 36 (NIR equipment included)	[44]
Vaz et al., 2018	Portugal	ICG + RI + BD	retrospective study	232	94% breast cancer cN0 and 6% post NACT	IR of ICG 91.4% IR of RI 97.2% IR of BD 89.5% In obese patients, no significant difference regarding IR between ICG, RI and BD	[48]
Yuan et al., 2018	China	ICG + BD vs. RI + BD	randomized trial	471	cTis-3N0	IR of ICG + BD 99% IR of RI + BD 99.6% FNR for ICG + BD and RI + BD were 5.6% and 7.5%, respectively (*p* > 0.05)	[43]

ICG: indocyanine green, BD: blue dye, RI: radioisotope, IR: identification rate, NACT: neoadjuvant chemotherapy, FNR: False negative rate, SLNB: sentinel lymp node biopsy.

## 5. ICG as SLNB Tracer in the Post NACT Setting

Over the past few years, due to the increasing tendency to omit ALND, the indications for performing SLNB for axillary staging in breast cancer have expanded beyond clinically node-negative patients. The current NCCN guidelines recommend that highly selected patients with biopsy proven and marked by clip lymph node metastases, who become clinically node-negative after primary systemic therapy, may undergo SLNB [4]. In these cases, in order to reduce the rate of false negatives (FNR), the NCCN panel recommends the use of a dual tracer, the removal of the clipped nodes and the excision of at least three SLNs, the so-called targeted axillary dissection (TAD).

ICG may prove to be useful within this context by increasing the success and by reducing the false negatives of SLNB in patients undergoing SLNB in the post NACT setting. The existing literature on the use of ICG after receiving NACT suggests that ICG is indeed effective in this context, and it may also enhance the success of SLNB. Jung et al. from Korea, in a phase 2 clinical trial, randomized 130 patients who received NACT to SLNB using either ICG plus RI, or RI alone as lymphatic mapping tracers [32]. The authors reported IR = 98.3% for the dual ICG+ RI method, compared with IR = 93.8% for the Technetium 99m alone method, and without significant difference between the two groups, regarding the mean number of SLNs retrieved.

A retrospective double-arm study by Staubach et al. evaluated the success of the ICG plus RI SLNB technique in patients in the post NACT setting, compared with early breast cancer patients receiving upfront SLNB. The authors found that the IR of ICG is 95.4% in patients after primary systemic therapy, and 94.3% for primary SLNB [37]. This is in accordance with the study by Jin et al. on the use of the triple ICG + RI + BD technique, that also included clinically node-negative patients as well as patients receiving NACT, and reported that the IRs were more than 98% and not significantly different between the two groups [51]. Another study by Papathemelis et al., a single-arm analysis on the use of dual ICG + RI method, included 104 patients, of whom 19 were in the post NACT setting, and reported an IR = 100% for the combined ICG + RI technique, with an FNR of 4.8% for RI in contrast to 0% for ICG. Interestingly, all of the patients that had received primary systemic therapy were ICG positive [36].

Two studies examined the efficacy of the dual ICG + BD technique in patients after NACT. Pinto et al. conducted an observational study in clinically node-positive patients who became node-negative after NACT and underwent SLNB-TAD and reported an IR = 89.2% for ICG and 85.5% for BD [17]. Similarly, Ng et al. also evaluated the dual ICG + BD technique and reported that the nodal detection rate was 87.5% after NACT and 100% after neoadjuvant endocrine therapy [21].

Evidence-based data suggest that SLNB in the post NACT setting is considered controversial because of the increased false negative rates that have been observed [53], which is why guidelines recommend the use of the standard dual RI + BD technique in these cases [4]. Therefore, it is reasonable to hypothesize that patients in these circumstances may benefit from adding ICG to the standard dual technique. Accordingly, Chirappapha et al., in his study of patients who underwent SLNB after NACT, reported that the FNR for ICG, BD and RI were 10%, 30% and 40%, respectively, whereas for the combined ICG + RI + BD method, the FNR drops below 10% [50].

## 6. ICG as SLNB Tracer in Obese Patients

Obesity has been reported to affect the outcome of both RI- and BD-guided SLNB in early-stage breast cancer patients, especially in older patients [54]. Evidence suggests that ICG may also be less effective as an SLNB tracer in obese patients. Lin et al., who studied the use of the combined ICG + BD method in early breast cancer patients, reported that an IR≈100% when the BMI < 30, whereas the IR drops below 75% in patients with BMI > 30 [22]. This is in accordance with another study by Takemoto et al. on the use of ICG + BD in upfront SLNB for early clinically node-negative breast cancer, which also reported significantly lower IR of ICG in overweight patients with BMI > 25 [30]. Similarly, the study on the use of ICG combined with RI by Mazouni et al. concluded that obesity strongly correlates with ICG failure [41]. On the contrary to the above studies, a prospective trial from the UK that enrolled all SLNB eligible breast cancer patients who underwent combined ICG + BD-guided SLNB found that ICG is effective irrespective of the patients BMI [21]. Finally, the retrospective study by Vaz et al. on the combined triple use of ICG + RI + BD for SLNB reported that there was no statistically significant difference regarding the performance of either of the three methods in comparison with the patient’s BMI [48].

## 7. ICG as SLNB Tracer within Clinical Practice Guidelines

The dual Technetium 99mm+ blue dye technique is long-established and is widely used as the gold standard method of lymph node mapping during SLNB. ICG fluorescence has emerged as a reliable alternative method, but consensus regarding its optimal role as a tracer is missing. In general, American guidelines have not yet included ICG as an option for SLNB mapping, whereas early on, clinical practice guidelines from Asia, and subsequently some guidelines from Europe, integrated ICG within the list of commonly used tracers in SLNB.

The American Society of Breast Surgeons in their Performance and Practice Guidelines for SLNB, recommend the use of the dual RI + BD method, since its success is superior to that of either technique alone [3]. Similarly, the American Society of Clinical Oncology, in their clinical practice guideline update, refers only to the radiotracer and the blue dye technique as SLNB tracers and does not mention novel SLN tracers [55].

On the other side of the Atlantic, in the recent evidence-based recommendations for health care in England from the National Institute for Health and Care Excellence (NICE), physicians are encouraged to perform SLNB using the dual technique with isotope and blue dye, again without any mention of novel tracers [7]. On the contrary, in the recent ESMO clinical practice guidelines, it is mentioned that with appropriate training in the dual RI + BD or ICG fluorescence technique, IRs of over 97%, low FNR and low axillary recurrence rates after SLNB are feasible [56].

The Japanese Breast Cancer Society in their recent clinical practice guidelines recommends the use of ICG fluorescence as a safe alternative to the use of radioisotope for the precise identification of the SLNs [57]. In accordance with this, the Chinese Society of Breast Surgery notes that, in China, isosulfan blue and patent blue are not approved, whereas the clinical application of radioisotopes is strictly managed and therefore difficult to be used widely [58]. For these reasons, in cases when the widely recognized RI/methylene blue technique is not available, the Chinese Society of Breast Surgery recommends the use of dual ICG+ methylene blue method, to improve the identification rate compared with the use of blue dyes alone [58]. Furthermore, the National Health Commission of the People’s Republic of China, in their recent guidelines, marks that the commonly used tracers for SLNB include RI, BD and hybrid tracers that combine a radioactive and a fluorescent molecule (such as ICG-99mTc-nanocolloid) [59]

## 8. Meta-Analyses on the Use of ICG as SLNB Tracer for Breast Cancer

Since the literature on the use of ICG for lymphatic mapping in breast cancer is constantly expanding, several meta-analyses have examined the effectiveness and accuracy of ICG compared with conventional SLNB tracers (Table 4).

One meta-analysis evaluated the use of the combined ICG + BD method, compared with the use of BD alone. Specifically, Wang et al. included 11 studies with 2137 patients in his meta-analysis and concluded that the combined ICG + BD technique is characterized by a significantly higher detection rate and by a significantly higher number of SLNs detected, compared with the use of BD alone [60].

Two meta-analyses evaluated the use of ICG in comparison with that of RI. Specifically, Sugie et al. included 12 studies with a total of 1736 patients and did not find a significant difference between the use of ICG and that of RI, in terms of the SLN IRs [61]. Regarding the detection of metastatic SLNs, the authors reported a trend towards better axillary staging in favor of ICG [61]. Accordingly, in the meta-analysis by Goonawargena et al., which included 19 studies with 2301 patients, ICG was found to be equivalent to RI regarding the SLN identification rate and the detection of tumor positive SLNs [62]. However, it should be noted that the combined use of ICG+ RI was found to be superior to the use of ICG or RI alone [62].

Seven meta-analyses examined the accuracy of the use of ICG, alone or combined with the conventional SLNB tracers, and compared it with the use of the conventional SLNB tracers. Kedrzycki et al. and Thongvitokomarn et al., in their meta-analyses that included 944 and 4216 patients, respectively, concluded that ICG is equivalent to RI and superior to BD regarding the SLN identification rates [63,64]. Rocco et al. and Yin et al., in their meta-analyses that included 3980 and 2671 patients, respectively, reported that ICG is a significantly better tracer compared with conventional single tracers (RI alone or BD alone) [65,66]. Regarding the comparison between ICG and the standard dual RI + BD technique, two meta-analyses concluded that ICG alone is comparable as a tracer with the standard dual mapping [66,67]. Furthermore, ICG was reported to be even better than the traditional dual RI + BD method by two other meta-analyses studies regarding the SLN’s identification rates [63,65]. Finally, the combined ICG + BD method, as well as the combined ICG + RI method, can both be used as effective mapping methods, alternative to the standard dual RI + BD technique [68,69].

**Table 4:** Meta-analyses on the use of ICG as SLNB tracer in breast cancer.

Meta-Analysis	Tracers	No. of Studies	No. of Patients	Results	Reference
Wang et al., 2023	ICG + BD vs. BD	11	2137	ICG + BD has significantly higher detection rate, and significantly higher number of SLNs detected, compared with BD alone	[60]
Rocco et al., 2023	ICG vs. conventional tracers	22	3980	ICG has significantly higher identification rate and significantly higher number of SLNs detected, compared with conventional single or dual tracer	[65]
Liu et al., 2021	ICG + BD vs. RI + BD vs. BD	49	7498	Both ICG + BD and RI + BD can be used as effective mapping methods to improve the detection rate, compared with BD alone For ICG + BD the IR is 97% and FNR 7% For RI + BD the IR is 96% and FNR is 7%	[68]
Yin et al., 2020	ICG vs. BD ICG vs. RI ICG vs. RI + BD	21	2671	ICG is better tracer compared with BD or RI alone, and is not a worse tracer compared with BD + RI	[66]
Kedrzycki et al., 2021	ICG vs. BD ICG vs. RI ICG vs. RI + BD	10	944	ICG is equivalent to RI regarding SLN identification, and superior to BD alone and to gold standard BD + RI	[63]
Thongvitokomarn et al., 2020	ICG vs. BD ICG vs. RI	30	4216	ICG is equivalent to RI regarding SLN identification, and superior to BD The mean number of SLNs removed were 2.35, 1.92 and 1.72 for ICG, BD and RI, respectively	[64]
Goonawardena et al., 2020	ICG vs. RI ICG + RI vs. RI ICG + RI vs. ICG	19	2301	ICG is equivalent to RI regarding SLN detection and sensitivity (metastatic SLNs detection). Dual mapping with ICG + RI is significantly better compared with RI or ICG alone.	[62]
Mok et al., 2019	ICG vs. RI vs. BD vs. (RI + BD) vs. SPIO vs. CEUS	35	4244	ICG alone is superior to BD alone and comparable to the standard dual-technique of RI + BD, in terms of IR and FNR	[67]
Sugie et al., 2017	ICG vs. RI	12	1736	No significant difference between ICG and RI in terms of SLN IR	[61]
Niebling et al., 2016	ICG vs. (ICG + RI) vs. (RI + BD) vs. RI vs. BD	154 (88 breast cancer and 66 melanoma)	44,172	Regarding the included breast cancer studies: - IR of ICG, ICG + RI, RI + BD, RI, BD were 95%, 96%, 95%, 94% and 85%, respectively - FNR of ICG, ICG + RI, RI + BD, RI, BD were 2.8%, 0.1%, 1.5%, 2.2% and 3.2% respectively - Mean number of SLNs per patient for ICG 2.85, for ICG + RI 2.87, for RI + BD 1.89, for RI 2.17 and for BD 2.09	[69]

ICG: indocyanine green, BD: blue dye, SLN: sentinel lymph node, RI: radioisotope, IR: identification rate, FNR: false negative rate, SPIO: supermaramagnetic iron oxide, CEUS: contrast-enhanced ultrasound imaging.

### Unsolved Questions and Future Perspectives of ICG as SLN Tracer

ICG has long been discovered and it was first used for SLN mapping in breast cancer as a near-infrared fluorescent agent in 2005 [70]. Since that time, ICG has been proven to be effective as a fluorescent SLN tracer in breast cancer, but it has not become the standard of care. This is partly due to some limitations associated with this technique, as well as due to the poorly defined optimization and standardization of this method. ICG molecules are water soluble, but relatively hydrophobic, and tend to aggregate, resulting in reduced NIR fluorescence emission. This is the so-called aggregation-caused quenching (ACQ) emission of ICG [71]. When injected intravenously, ICG bounds to plasma proteins and it is rapidly extracted by the liver and excreted in the bile. After administration outside blood vessels (e.g., as an SLN tracer), ICG binds to proteins and is found within the lymph, reaching the closest lymph nodes normally within a few minutes. The ICG aggregation-caused quenching (ACQ) phenomenon is the reason why higher concentrations of ICG do not produce increased fluorescent emission. In the context of SLN mapping for breast cancer, the optimal concentration of diluted ICG and the optimal solvent for the solution are poorly defined in the literature. The variations across different studies regarding the injection technique, including the different ICG dose, the solvent used and the location for the injection (intradermal or subdermal, subareolar or periareolar or peritumoral) aim to overcome the challenge of the instability of the ICG pharmacokinetics from the injection site until reaching the target sentinel nodes. A concentration of <5 mg/mL and an injected volume of >2 mL have been reported to increase the detection rate, according to a recent meta-analysis [72]. Furthermore, instead of using saline, the dilution of ICG with colloids, including human serum albumin (HSA) and, lately, Voluven, has been reported to decrease the diffusion of ICG to the surrounding tissues and enable a more stable mapping quality of the lymphatic pathway and lymph nodes [71,73].

One major advantage of the use of ICG fluorescence for SLN mapping in breast cancer is the real-time visualization of the lymphatic pathway(s) of lymph drainage from the breast towards the axilla. This correlates with the low diameter of the molecule of ICG that is capable of easily and rapidly travelling through the lymphatic vessels [74]. The clear real-time image (before the skin incision) of the lymphatic vessels and the accurate positioning of the SLN is in contrast with the relative blindness that characterizes the search for SLNs with the use of blue dye or radioisotope. However, the low molecular weight of ICG might probably also correlate with the aforementioned increased number of lymph nodes retrieved with this method. Specifically, the small molecule of ICG may readily diffuse through the true SLNs towards other adjacent lymph nodes [73]. One could also hypothesize that the lower diameter of ICG may also facilitate its diffusion through lymphatic channels that are partially blocked by neoplastic or inflammatory cells. Interestingly, it has been reported that the detection with ICG of multiple routes from the breast towards the axilla is more common in breast cancer cases with lymph node skip metastases [75]. Overall, it is unclear why ICG seems to be superior regarding the identification rates and the false negative rates, compared with the conventional tracers. Furthermore, it should be emphasized that in most of the studies included in this review, the authors most commonly chose to use ICG in combination with the conventional tracers. Thus, caution is needed in evaluating the results of the above studies, since the performance of each method might be affected by the concurrent presence of other tracers within the lymphatic routes. Randomized controlled trials comparing the use of ICG alone with the use of conventional tracers would undoubtedly reach more reliable conclusions.

One significant drawback of the use of ICG fluorescence is the contamination of the surgical field, when lymphatic vessels emitting fluorescent signals are damaged, resulting in ICG leakage into the surrounding tissues, thus impairing the accurate detection of SLNs. Some authors suggest clipping the lymphatic channels before the removal of the SLNs, in order to avoid the leakage of the fluorescent agent [52]. Furthermore, in case of ICG leakage, the concurrent use of a second tracer (blue dye or radiotracer) may facilitate the SLN detection [27].

Regarding the future implementation of ICG fluorescence in SLN mapping in breast cancer, one must point out that there is a clear trend towards ensuring surgical accuracy in the era of precision medicine. As a consequence, the development of hybrid radioactive and fluorescent tracers may prove to be clinically significant. By using hybrid tracers, the target SLNs can be accurately localized irrespective of the depth from the skin (guided by the radiotracer) and resected under real-time clear optical navigation (guided by the fluorescent agent). Furthermore, the use of hybrid tracers eliminates the discrepancies observed when using multiple tracers concurrently. One such example of hybrid tracer is ICG-99mTc-nanocolloid that has been reported to be a successful tracer for image-guided SLN biopsy in breast cancer [76,77].

## 9. Conclusions

The conventional tracers for SLN mapping in breast cancer have some disadvantages. Anaphylactic reactions to blue dyes and logistics issues related to radiotracers have led to the introduction of novel tracers into clinical practice. ICG is the most promising among them. The evidence so far suggests that ICG is superior to BD alone and at least comparable to RI alone, or to the standard dual BD + RI method. To date, there is no consensus regarding the optimal role of implementing ICG in the clinical practice of SLN mapping. Further research is needed in order to answer the question whether ICG should be used in combination with the conventional SLNB tracers, or if ICG could potentially replace them. Patients undergoing SLNB in the post NACT setting may benefit from triple mapping, with the addition of ICG to the standard dual RI + BD technique. Finally, the development of hybrid radioactive and fluorescent tracers that combine radio-guidance and optical guidance may also prove to be clinically significant as SLN tracers in breast cancer.
